# The association between high fructose corn syrup and the development of type-2 diabetes

**DOI:** 10.3389/fcdhc.2026.1785203

**Published:** 2026-03-17

**Authors:** Majid Almansouri

**Affiliations:** Department of Clinical Biochemistry, Faculty of Medicine, King Abdulaziz University, Jeddah, Saudi Arabia

**Keywords:** *de novo* lipogenesis, fructose, high fructose corn syrup, metabolism, policy control measures, type 2 diabetes

## Abstract

Fructose and high-fructose corn syrup (HFCS) have become central to the debate on metabolic health and the rising prevalence of type 2 diabetes. Chemically, fructose is a monosaccharide found naturally in fruits and honey, whereas HFCS is an industrially produced sweetener composed of varying proportions of free fructose and glucose. While fructose has unique metabolic effects, its impact is comparable to other sugars when consumed in excess. HFCS is widely used in processed foods and sugar-sweetened beverages (SSBs) due to its high sweetness and low production cost. However, its metabolic effects remain a topic of scientific and public health concern. Animal and human studies suggest that excessive fructose consumption contributes to metabolic disturbances, including insulin resistance, impaired glucose tolerance, and increased fat accumulation in the liver through *de novo* lipogenesis (DNL). Unlike glucose, fructose bypasses key regulatory steps in glycolysis, leading to unregulated hepatic uptake and lipid synthesis. Epidemiological studies have reported a higher prevalence of type 2 diabetes in countries with greater HFCS availability, independent of obesity rates. Despite this, there remains controversy regarding whether HFCS is a direct contributor to diabetes or if overall energy intake plays a more significant role. This study aims to analyze the chemical composition of fructose and HFCS and their potential role in the development of type 2 diabetes. Additionally, it briefly discusses the global policy measures, such as sugar taxation and public health interventions, aimed at reducing sugar consumption and mitigating diabetes risk.

## Introduction

The increasing prevalence of metabolic syndrome and type 2 diabetes presents a major global health challenge. Metabolic health is increasingly at risk in societies where easy access to highly palatable, energy-dense foods and beverages is coupled with sedentary lifestyles and minimal physical activity (1). Recent data indicate that the global prevalence of type 2 diabetes has risen significantly. In 2024, about 589 million adults (20–79 years) globally were living with diabetes, corresponding to ~11.11% of the adult population, with prevalence projected to rise substantially by 2050 (2). This number is projected to surge to 650 million by 2030 and 780 million by 2045. ​ Nearly 58% of patients with diabetes reside in Asia, likely due to the genetic predisposition of the population in this region (3). The prevalence of diabetes also varies between low- and middle-income countries (LMICs) and high-income countries (HICs). In 2021, 3 in 4 adults with diabetes lived in LMICs. This disparity is expected to continue, with LMICs experiencing a more significant increase in diabetes prevalence compared to HICs (2). Ecological analyses have reported higher type 2 diabetes prevalence in countries with greater HFCS availability (4). However, such country-level comparisons cannot establish causality and are subject to important confounding factors, including overall consumption of ultra-processed foods, physical inactivity, and socioeconomic differences. ​Similarly, the prevalence of type 2 diabetes in Saudi Arabia is mainly influenced by factors such as unhealthy diets, low levels of physical inactivity, and urbanization (5). The World Health Organization ranks Saudi Arabia as having the second-highest diabetes rate in the Middle East (6). This emphasizes that type 2 diabetes is influenced not only by genetic factors but also by the nature of dietary habits and lifestyle choices.

A high consumption of added sugars, which is often present in a typical western diet – is considered to be a principal factor promoting metabolic derangements (7, 8). More precisely, excessive intake of sweetened soft beverages (SSBs) has been reported to be associated with a relatively higher risk of obesity and type 2 diabetes (9, 10). Studies on sugar intake in Europe, South America, and the United States found that mean sugar intakes in most countries were higher than the recommended intake (11). Particularly concerning is the rising consumption of fructose, sucrose and high-fructose corn syrup (HFCS), which have been directly associated with impaired glucose tolerance, high insulin resistance and metabolic disturbances (12). Type 2 diabetes prevalence is higher in countries with greater HFCS accessibility compared to those with lower HFCS access, regardless of obesity rates (4). Since evidence published in 2004 identified a temporal association between increased HFCS availability and obesity prevalence, a fructose-focused perspective on cardiometabolic disease has developed (9). Evidence has shown that fructose intake specifically has harmful effects on metabolic health and significantly increases the risk of impaired glucose tolerance (4, 13). Therefore, the main global challenge was concerned with the use of high fructose corn syrup (HFCS) in food and beverage production.

This study discusses the composition of “added sugars” particularly fructose and HFCS and their potential role in the development of type 2 diabetes. It also explores global policy efforts, including sugar taxation and public health initiatives, aimed at reducing sugar consumption and mitigating diabetes risk.

## Fructose and high fructose corn syrup composition

Fructose is commonly found in variety of non-alcoholic beverages like soft drinks and fruit juices, as well as processed foods including baked goods, flavored yogurts, condiments, sauces, jams, cereals, snacks, and desserts. Checking ingredient labels is essential, as fructose is frequently added to processed items due to its sweetness and low cost. Fructose has a greater sweetening capacity than glucose (14). However, both fructose and glucose naturally occur in fruits and honey as monosaccharides, whereas HFCS is produced industrially from corn starch through enzymatic isomerization of glucose to fructose, resulting in mixtures containing varying proportions of free fructose and glucose (1). HFCS, unlike sucrose “table sugar”, is a liquid sweetener derived from corn starch by enzymatic isomerization of glucose to fructose, which typically produces HFCS-42 (≈42% fructose) at equilibrium; higher-fructose syrups (e.g., HFCS-55 and HFCS-90) are obtained by additional enrichment/fractionation steps and subsequent blending to achieve the desired fructose-to-glucose proportions (15) (**Table 1**). It was developed in the 1960s as a liquid sweetener to replace sucrose, and it was introduced to the food and beverage industry in the 1970s. Based on its fructose content, HFCS is classified into HFCS 42, 55 and 90. HFCS-42 contain 42% fructose and 53% glucose, widely utilized as a sweetener in processed foods. HFCS-90 comprises 90% fructose and 10% glucose (15). It is used in small quantities for specialized applications but is primarily blended with HFCS to produce HFCS-55, which is used in beverage manufacturing (16). HFCS-55 contains 55% fructose, and 45% glucose mainly found in soft drinks and beverages. Currently, the availability of HFCS-42 is approximately balanced in the United States market (17). The relationship between fructose or HFCS and cardiometabolic diseases is influenced by their intake levels and the body’s metabolic response to these sugars. Therefore, its consumption in the United States has been declining due to increasing awareness of its potential health risks.

**Table 1:** The difference between different types of added sugars (sucrose. fructose, high fructose corn syrup, and glucose).

Feature	Sucrose	HFCS	Fructose	Glucose
Composition	50% glucose, 50% fructose (bound together)	42%-55% fructose, remainder glucose (free form)	100% fructose	100% glucose
Source	Sugar cane, sugar beets	Corn starch (enzymatic processing)	Fruits, honey, some vegetables	Carbohydrates (starches, grains, vegetables)
Structure	Disaccharide (fructose & glucose chemically linked)	Monosaccharides (fructose & glucose exist separately)	Monosaccharide	Monosaccharide
Sweetness	Moderately sweet	Slightly sweeter (especially HFCS-55)	Sweetest natural sugar	Less sweet than fructose
Digestion & Absorption	Requires sucrase enzyme for breakdown	Directly absorbed without enzymatic breakdown	Primarily metabolized in the liver	Absorbed in the intestines and used for energy
Metabolic Effects	Raises blood glucose & insulin response	Promotes liver fat accumulation, insulin resistance	Increases *de novo* lipogenesis (DNL), liver fat, insulin resistance	Stimulates insulin release and is primary energy source
Usage in Food Industry	Granulated sugar, baked goods, candies	Processed foods, soft drinks, condiments	Fruits, honey, added sugars in food	Natural carbohydrate metabolism, medical treatments

## Fructose metabolism

**Figure 1:**
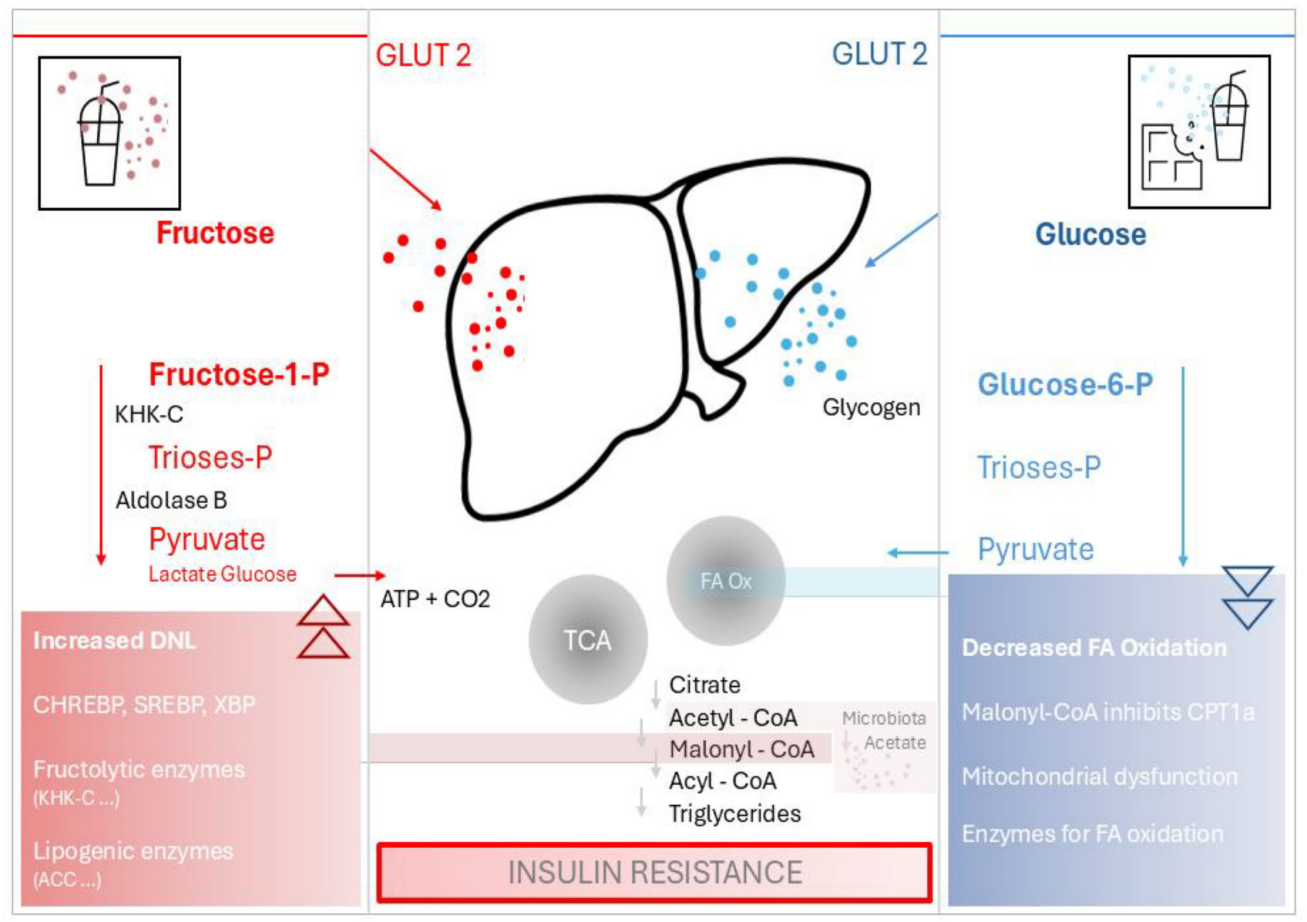
A comparison of hepatic metabolism of fructose and glucose following high sugar intake from SSBs, highlighting that increased *de novo* lipogenesis from fructose intake combined with reduced fatty acid oxidation contributes to hepatic fat accumulation. Key components include ACC, Acetyl-CoA-carboxylase; ATP, Adenosine triphosphate; CPT1a, Carnitine palmitoyltransferase 1A; FA, Fatty acid; GLUT, Glucose transporter; KHK-C, Ketohexokinase-C; Ox, Oxidation, Phosphate; SSB, Sugar-sweetened beverage; TCA, Tricarboxylic acid cycle.

Fructose absorption primarily occurs through facilitated diffusion via glucose transporter 5 (GLUT5), expressed on small intestinal epithelial cells (18, 19). GLUT proteins, encoded by the SLC2 genes, belong to the major facilitator superfamily of membrane transporters (20). They consist of approximately 500 amino acids, contain a single N-linked oligosaccharide, and feature 12 transmembrane domains. GLUT5 is primarily expressed in the small intestine and is also detected in tissues such as the kidneys, adipose tissue, and skeletal muscle; reported brain expression is limited and has not been demonstrated in neurons or astrocytes, while hepatic expression remains minimal. Unlike glucose, its transportation process does not involve ATP hydrolysis and functions independently of sodium absorption. When dietary fructose intake is low, a portion of it is converted directly into glucose by small intestinal enterocytes and then metabolized (21). In case of high fructose intake that cannot be digested in small intestines, they are transported into the liver for digestion (22). Upon entering circulation, fructose is predominantly extracted by the liver, the primary site of its metabolism (23). The kidneys, skeletal muscles, and adipose tissue also contribute to fructose metabolism in minor amounts (23). In contrary, glucose is mainly absorbed by the liver and muscle tissues, where it is stored as glycogen (24). Fructose, but not glucose, is phosphorylated in the liver by fructokinase (ketohexokinase; KHK), which exists in two isoforms: ketohexokinase-A (KHK-A) and ketohexokinase-C (KHK-C) (24). In contrast to glucose, which is metabolized through tightly regulated glycolytic pathways, fructose metabolism via KHK bypasses key regulatory steps, leading to unregulated hepatic uptake, rapid ATP depletion, and enhanced lipogenesis (24). KHK catalyzes the initial step of fructose metabolism by phosphorylating fructose into fructose-1-phosphate (F1P) (**Figure 1**). Indeed, excessive fructose breakdown through this pathway can result in ATP depletion (24).

KHK-C facilitates rapid hepatic fructose uptake by phosphorylating fructose without feedback inhibition, resulting in unregulated uptake (25). The expression levels of enzymes involved in fructose metabolism influence tissue-specific fructose handling. Ishimoto et al. found that KHK-C is highly expressed in hepatocytes and other tissues, rapidly phosphorylates fructose (25). Therefore, inhibition of KHK-C protects against fructose-induced metabolic disruptions (26). Park et al. demonstrated that fructose metabolism via KHK-C induces endoplasmic reticulum stress, worsening fatty liver when combined with a high-fat diet, while KHK knockdown improves metabolic function and reduces liver injury in both mice and human studies (27).

Once fructose is phosphorylated, it is metabolized into lactate or converted to other sugars such as glucose, glycogen, or lipids, which stimulate *de novo* lipogenesis (DNL) (1). Nonetheless, moderate consumption of fructose-containing SSBs causes additional abnormal metabolic changes, including reduced hepatic insulin sensitivity and increased hepatic lipogenic activity (1, 28). DNL converts excess carbohydrates, especially fructose, into fatty acids, which are then stored as triglycerides or transported to adipose tissue for storage. Fructose also bypasses the regulatory enzyme phosphofructokinase, entering glycolysis unregulated and thus promoting DNL, increase the production of acetyl-CoA, the building block for fatty acid synthesis (1, 29). The excess acetyl-CoA generated from fructose metabolism is channeled into the DNL pathway. As a result, fructose can trigger epigenetic modifications and metabolic changes that redirect calories toward storage within abdominal fat cells (30). The transcription factors ChREBP (Carbohydrate Response Element-Binding Protein) and SREBP-1c (Sterol Regulatory Element-Binding Protein 1c) are upregulated, enhancing the expression of lipogenic enzymes such as acetyl-CoA carboxylase (ACC) and fatty acid synthase (FAS) (26, 31). This leads to the synthesis of triglycerides and other lipids, contributing to hepatic fat accumulation.

Elevated hepatic fat content increases fatty acid (FA) supply for triglyceride synthesis while inhibiting FA oxidation (32). Subsequently, the hepatic insulin resistance increase, leading to impaired glucose regulation and elevate circulating triglyceride in the blood. These consequences increase risk of metabolic syndrome, particularly type 2 diabetes and non-alcoholic fatty liver disease (NAFLD) (33). It also underlines that high levels of DNL-derived fatty acids strongly predispose individuals to other features of metabolic syndrome (34). Moreover, fructose may contribute to peripheral insulin resistance in skeletal muscle by stimulating excessive hepatic free fatty acid production, increasing free fatty acid release from very-low-density lipoproteins, and promoting lipid accumulation within muscle cells (13). Excessive fructose intake in rodents was shown to cause full metabolic syndrome associated with peripheral insulin resistance (34). However, moderate to low dietary fructose can disrupt hepatic glucose regulation but does not immediately induce muscle insulin resistance.

## Mechanistic pathways linking fructose and HFCS to metabolic dysfunction

Fructose and HFCS promote metabolic dysfunction through convergent nutrient-sensing and lipogenic pathways. Hepatic fructose metabolism bypasses key glycolytic control points, leading to substrate overload and activation of DNL (35). This process is transcriptionally regulated by ChREBP and SREBP-1c, which upregulate lipogenic enzymes and triglyceride synthesis. Fan et al. demonstrated that amino acid–mTORC1 signaling phosphorylates PDX1 at serine-61, increasing its protein stability and transcriptional activity (36, 37). This modification enhances β-cell proliferation and insulin expression and contributes to diet-induced hyperinsulinemia, obesity, and hepatic steatosis (37). In parallel, fructose has been shown to activate mTOR signaling, a central nutrient-sensing pathway that integrates carbohydrate availability with anabolic metabolism, insulin signaling, and lipid synthesis (35). Dysregulated mTOR activity further amplifies hepatic lipogenesis and suppresses fatty acid oxidation, supporting insulin resistance and ectopic fat accumulation. Collectively, these interconnected pathways provide a mechanistic framework linking excessive fructose or HFCS intake to metabolic derangements, particularly under conditions of positive energy balance.

## The association between HFCS and the development of type 2 diabetes

Developed using glucose isomerase technology in the 1960s and introduced commercially in the United States in the 1970s, HFCS is an inexpensive sweetener widely used by the food and beverage industries (36, 37). Between the 1970s and 2000s, the annual consumption of HFCS by Americans surged significantly from approximately 0.25 kg to 30 kg. During the same period, sucrose intake gradually declined from around 45 kg to 30 kg, while daily fructose consumption rose by about 25% (17). Epidemiological and experimental studies on animal and human have revealed the fundamental relationship between HRCS and metabolic diseases including obesity, and type 2 diabetes. Among the sugars, HFCS accessibility has individually predicted greater diabetes prevalence, even when adjusting for obesity and total sugar and calorie availability (4). While HFCS consumption is acknowledged as a risk factor for type 2 diabetes and high triglyceride level, the specific role of fructose in the development of these diseases remains a topic of ongoing debate. On the other hand, some studies suggest that fructose-containing sugar intake is not linked to the development of type 2 diabetes or obesity (38–40). These conflicts have led to ongoing debate about whether HFCS is a direct risk factor for type 2 diabetes, or if excessive energy intake is the primary cause.

Animal studies have provided understandings into mechanisms underlying metabolic differences between glucose and fructose and the metabolic effect of HFCS in developing type 2 diabetes (41). The applicability of animal models to humans is limited by their use of fructose doses and inherent differences in carbohydrate metabolism between species (29). While animals can derive over 50% of their fatty acids from DNL, this percentage is considerably lower in humans (17). A key area of investigation involves abnormal gene expression associated with sugar metabolism. Mice lacking the transcription factor ChREBP exhibit reduced expression of lipogenic enzymes (31). ChREBP deletion redirects excess carbohydrate metabolism toward glycogen storage, increasing hepatic glycogen content and reducing liver fat. Notably, fructose stimulates hepatic ChREBP and its downstream targets more effectively than glucose (26). Another gene responsive to sugar intake is SREBP-1c, which is strongly induced by high-fructose diets through insulin and TOR signaling pathways (42). Tong et al. identified E4BP4 as a key regulator of hepatic *de novo* lipogenesis, stabilizing SREBP-1c and enhancing lipid synthesis in response to insulin during the fed state (43).

While HFCS has been suggested as a contributor to obesity, studies have shown that excessive intake of 25% HFCS can increase body weight, fat mass, and impair glucose tolerance in mice (44). However, some reports indicate that consuming SSBs does not necessarily result in obesity or secondary diabetes (33). Hidaka et al. investigated the impact of excessive HFCS intake under energy restriction on type 2 diabetes development in middle-aged mice (45). Despite no obesity or lipid changes, HFCS impaired glucose tolerance and reduced pancreatic weight, suggesting metabolic dysfunction even without weight gain, with potential age-dependent effects in humans. Hattori et al. examined the impact of excessive HFCS consumption on glucose tolerance and obesity in mice under controlled caloric intake (46). Despite unchanged body weight, HFCS intake impaired glucose tolerance due to insulin secretion defects, suggesting that excessive HFCS consumption contributes to type 2 diabetes risk, even without obesity. Interestingly, Stanhope et al. evaluated how beverages sweetened with high fructose influenced body fat distribution, measuring subcutaneous, visceral, and abdominal fat (47). After 10 weeks, diets enriched in fructose were associated with increased visceral abdominal fat and impaired glucose tolerance. The study emphasized the metabolic effects of fructose itself rather than commercially formulated HFCS, which contains both fructose and glucose (12).

A study by Schwarz et al. on human subjects has compared the effect of HFCS, provided through beverages, against an isocaloric diet with identical macronutrient composition but with compound carbohydrates in solid form (48). After nine days, the diet with high fructose was associated with increased hepatic DNL. Nonetheless, fructose consumption through soft drinks is associated with higher cardiometabolic factors such as high fasting blood glucose, and hyperlipidemia causing secondary diabetes (49). On the other hand, other dietary sugars that do not contain fructose appear to have fewer harmful effects. For instance, a six-month randomized trial in overweight individuals found that, unlike isocaloric milk, diet soda, and water, sucrose-sweetened sodas specifically led to a minimal increase ectopic fat accumulation and elevated lipid levels (50). A study comparing the effects of a eucaloric diet where 25% of calories came from fructose versus glucose on energy-regulating hormones—insulin, leptin, and ghrelin—found that fructose resulted in lower insulin and leptin increases and less ghrelin suppression than glucose, potentially promoting increased Energy intake (51). A meta-analysis of human studies revealed that increasing consumption of fructose from processed foods and beverages is associated with higher fasting blood glucose levels (52).

Global epidemiological studies on human subjects relating HFCS with type 2 diabetes were fairly described in the literatures. Goran et al. examined global relationships between HFCS availability and type 2 diabetes prevalence across 43 countries. Diabetes prevalence was 20% higher in countries with greater HFCS availability, independent of obesity, total sugar, calorie intake, BMI, population, and GDP, suggesting HFCS uniquely impacts diabetes risk at the population level (4). None of the existing cohort studies have identified a clear positive link between total sugar intake and type 2 diabetes. However, associations between specific sugar types and the development of diabetes or metabolic diseases have been observed in various cohort studies. Soft drink and juice consumption containing fructose or HFCS were found to increase risk of type 2 diabetes in Chinese Singaporeans (53). A 43,580-participant study found higher diabetes incidence with ≥2 weekly servings, especially in those gaining ≥3 kg. SSBs significantly raise diabetes risk, while artificially sweetened drinks show weaker associations due to confounding health factors (54). A 16-year study of 43,960 African American women participants found a higher diabetes risk with increased intake of soft drinks and fruit drinks, emphasizing the overlooked impact of fruit drinks on diabetes development (12). Additionally, there was also a Borderline positive association between HSCS, and type 2 diabetes (p=0.01) found by Montonen et al. (10). In the United Kingdom, a study examined the relationship between different SSBs, artificially sweetened beverages, and fruit juice consumption with type 2 diabetes risk (55). Findings from 25,639 adults revealed that soft drinks and sweetened-milk beverages were positively associated with type 2 diabetes, while sweetened tea/coffee and fruit juice were not. Substituting artificially sweetened beverages for sugar-sweetened drinks did not significantly lower diabetes risk.

More interestingly, 100% fruit juice, although not classified as a sugar-sweetened beverage, contains free sugars that are no longer embedded within the intact fiber matrix of whole fruit. While several meta-analyses have reported no association or only a very weak association between moderate 100% fruit juice consumption and type 2 diabetes risk (56), excessive intake has been linked to weight gain and adverse glycemic outcomes in some observational studies (57). This metabolic response differs from that of sugar-sweetened beverages, which provide added sugars without accompanying nutritional or bioactive components, whereas whole fruit consumption is not associated with these adverse effects (56).

## The association of HFCS with diabetic complications

HFCS has been linked not only to diabetes- related metabolic abnormalities but also to end- organ damage and diabetic complications (58). Isolated fructose or HFCS has been shown to cause renal injury in animal and human studies (59, 60). In rodents, chronic isolated fructose intake is associated with diffuse effect on proximal tubules, whereas starch-fed counterparts do not develop this renal abnormality or diabetic microangiopathy observed in fructose-fed rodents (61). Lanaspa et al. explored the role of endogenic fructose metabolism in patient with diabetic nephropathy (62). In diabetic mice, fructokinase activation led to rapid phosphorylation of fructose with intracellular phosphate trapping, resulting in phosphate depletion and impaired ATP resynthesis, thereby promoting ATP depletion, inflammation, and renal injury (63). Fructokinase-deficient mice exhibited reduced kidney damage, highlighting the pathogenic role of endogenous fructose production via the polyol pathway—where glucose is converted to sorbitol by aldose reductase and subsequently to fructose by sorbitol dehydrogenase—rather than dietary fructose alone, in the progression of diabetic nephropathy. Andres-Hernando et al. further identified fructokinase-driven phosphate depletion as a central mechanism in ischemic acute kidney injury, with fructokinase inhibition significantly reducing ATP depletion, oxidative stress, inflammation, and renal damage in affected mice (63). Another study also found that high fructose intake worsened renal lesions in Spontaneously Diabetic Torii (SDT) rats, primarily affecting renal tubules and interstitial tissues, likely due to increased uric acid and blood glucose levels from excessive fructose consumption (64).

Additionally, fructose alone contributes to other diabetes-related microvascular complications, including impaired motor nerve conduction velocity (neuropathy) (65). Garcia et al. investigated fructose-induced insulin resistance as a model for neuropathic pain (66). Chronic fructose consumption increased insulin levels, induced hyperalgesia, and altered ion channel expression in dorsal root ganglia and sciatic nerve. Metformin treatment partially reversed these effects, highlighting fructose’s role in neuropathic pain through insulin resistance and neuronal modulation. Postprandial fructose levels are also linked to retinopathy in type 2 diabetes patients (67). Furthermore, in a review by Delbridge et al., the link between HFCS and cardiomyopathy was explored, highlighting fructose’s harmful effects on cardiomyocytes, especially in diabetic patients, causing significant heart failure (68). Fructose metabolism in cardiomyocytes is facilitated by proteins enabling fructose transport and utilization. Elevated dietary fructose intake and insulin resistance promote unregulated glycolysis and oxidative stress, contributing to cardiac injury. Additionally, fructose’s high reactivity accelerates harmful protein modifications like O-GlcNAcylation and advanced glycation end-product formation. The review underscores the need for more research to clarify fructose’s cardiopathogenic effects. Isolated fructose feeding in rats has been also shown to induce arterial atherogenesis (69).

## Fructose restriction policy

Large-scale cohort studies and meta-analyses have consistently reported that regular consumption of one or more SSBs per day is associated with an approximately 18%–26% increased risk of type 2 diabetes (70). In some cohorts, higher risk estimates have been reported; for example, individuals consuming more than one SSB daily had up to an 85% higher risk of developing type 2 diabetes in an 8-year prospective study (70). While it is widely acknowledged that excessive sugar consumption should be avoided, debates persist regarding the appropriate upper limit. The World Health Organization (WHO) recommends capping free sugar intake at 10% of total calories, with an ideal target of 5% (71). The American Heart Association (AHA) proposes even stricter limits, advising no more than 150 kcal of added sugars per day for men and 100 kcal for women (8). The Institute of Medicine (IOM) defined an upper intake level of up to 25% of total energy from added sugars within the acceptable macronutrient distribution range, a threshold intended to ensure adequate nutrient intake and prevent deficiency rather than to represent a health-optimizing recommendation (72). Although the AHA’s Scientific Statement acknowledges the limited availability of prospective trial data, its recommendations are largely based on observational studies, particularly those examining high SSBs consumption. Notably, many adverse metabolic effects—such as insulin resistance, hyperinsulinemia, hypertriglyceridemia, and hypertension—can be reversed by reducing added fructose intake (73, 74).

Several dietary guidelines have been established to address sugar intake, with most emphasizing the need to reduce added fructose-containing sugars to support a healthy body weight. Maintaining a balanced macronutrient intake that includes moderate levels of fats and carbohydrates, while limiting sugar consumption, is considered essential for a nutritious diet. Notably, free sugars encompass both monosaccharides and disaccharides that are intentionally added to foods and drinks, along with sugars naturally occurring in honey, syrups, fruit juices, and fruit juice concentrates (75). Although fructose produces a lower immediate postprandial glycemic response than glucose, contemporary diabetes management guidelines do not recommend the use of fructose as an added sugar (76, 77). Excessive fructose intake may elevate triglyceride levels, promote insulin resistance, and affect overall metabolic health. Modern diabetes care emphasizes total energy balance and cardiometabolic risk rather than isolated glycemic responses (76, 77). The strategies involve all fructose-containing sugars. Further studies examined the role of DNL in fructose-induced hypertriglyceridemia and whether physical activity can mitigate these effects. Egli et al. compared healthy subjects on either low-fructose, high-fructose/low-activity, or high-fructose/high-activity diets (77). Increased fasting and postprandial triglycerides occurred with high-fructose intake but were prevented by physical activity, demonstrating exercise protects against fructose-induced triglyceridemia. A study by Macedo et al. investigated how aerobic exercise impacts postprandial lipemia (PPL) following fructose intake (78). Results showed that prior exercise reduced triglyceride levels by approximately 30% when measured 13 hours later but not after 37 hours. Regular exercise appears essential for maintaining consistent hypolipemic effects. Bartolotti et al. also investigated how high protein intake affects lipid oxidation and postprandial triglycerides after fructose ingestion (79). Their study found that while high protein meals increased energy expenditure, they inhibited lipid oxidation and enhanced fructose-induced gluconeogenesis. Notably, high protein diets did not improve lipid oxidation but increased postprandial triglycerides in individuals on hyper-energetic, high-fructose diets.

Prohibiting the sale of SSBs in public schools, limiting their availability on university campuses, and restricting their purchase through the Supplemental Nutrition Assistance Program reflect broader policy efforts to curb excessive sugar consumption, particularly among children and young adults, as part of the National School Lunch Program (80). McElrath et al. examined SSB availability in US secondary schools from 2007-2009, highlighting that non-soda SSBs were the most accessible (81). Policies allowing beverage suppliers’ influence increased SSB access, while comprehensive nutritional guidelines reduced availability. Effective interventions should target both soda and non-soda SSBs and minimize supplier involvement. These initiatives aim to reduce the accessibility of SSBs in educational environments where consumption habits are formed.

Beyond school-based policies, a growing international movement advocates for taxation on SSBs to discourage consumption and generate revenue for public health programs addressing obesity and metabolic diseases (82). Countries such as Mexico, the United Kingdom, and South Africa have implemented sugar taxes, leading to reduced sales of high-sugar beverages and prompting reformulation by manufacturers (82). In Canada, the Childhood Obesity Foundation has pushed for stricter marketing restrictions on SSBs, recognizing the influence of advertising on children’s dietary choices. Additionally, the foundation has proposed “smart taxation” policies, directing funds from SSB taxes toward obesity prevention programs, public health campaigns, and nutritional education initiatives (83). These measures reflect a broader strategy to combat rising obesity rates by addressing the role of added sugars in the diet and promoting healthier alternatives.

In Saudi Arabia, significant measures have been implemented to curb the consumption of SSBs due to their association with obesity, type 2 diabetes, and dental caries. In June 2017, the Kingdom introduced a 50% tax on soft drinks and a 100% tax on energy drinks. This taxation was further expanded in December 2019 to include all sweetened beverages with a 50% levy (6). These fiscal policies aim to reduce the intake of added sugars among the population, thereby addressing the high prevalence of obesity and diabetes in the country. While these taxes have led to price increases, their impact on actual consumption patterns, particularly among children, has been mixed. For instance, a study in the Eastern Province observed an 8% reduction in energy drink consumption but a 2% increase in soft drink consumption among schoolchildren post-tax implementation (6). This suggests that taxation alone may not be sufficient and should be complemented by public health campaigns to effectively reduce SSB consumption and improve health outcomes.​ Another recent study by Alzaben et al. examined soft drink consumption patterns in Saudi Arabia five years after tax implementation (84). Among 1,935 adults, 83% consumed soft drinks monthly, mainly due to habit and social gatherings. Availability, affordability, and gatherings influenced consumption, highlighting the need for additional strategies beyond taxation to reduce intake effectively.

It is important to acknowledge that not all evidence supports a uniquely harmful metabolic effect of fructose independent of total caloric intake. Several controlled human studies and meta-analyses have shown that when fructose is consumed under isocaloric conditions, its effects on body weight, glycemic control, and insulin sensitivity are often comparable to those of other carbohydrates. These suggest that excess energy intake and dietary context play a major role in mediating fructose-related metabolic risk, particularly in clinical settings characterized by hypercaloric consumption.

## Conclusion

The growing prevalence of type 2 diabetes globally highlights the need to address dietary patterns that promote excessive energy intake, including high consumption of sugar-sweetened beverages and fructose-containing sweeteners such as high-fructose corn syrup (HFCS). While fructose and HFCS have been associated with insulin resistance, hepatic lipogenesis, hypertriglyceridemia, and fatty liver disease, these adverse effects are most consistently observed in settings of chronic caloric excess. In contrast, controlled isocaloric studies in which fructose replaces other carbohydrates often demonstrate attenuation or absence of these metabolic disturbances. Thus, HFCS should not be viewed as intrinsically toxic, but rather as a contributor to metabolic risk when consumed in energy-dense diets typical of modern food environments. Population-level strategies aimed at reducing excessive sugar intake, alongside broader lifestyle interventions, remain important for mitigating diabetes risk.
