# Supplementary material for: The association between high fructose corn syrup and the development of type-2 diabetes
Source: Front Clin Diabetes Healthc. 2026 Mar 17;7:1785203. doi: 10.3389/fcdhc.2026.1785203 (PMC13035500; doi:10.3389/fcdhc.2026.1785203)
Supplement: Supplementary file 1 [file Table1.docx]

# Supplementary Table. Summary of Evidence on Fructose/HFCS and Metabolic Risk in Type 2 Diabetes

| **Evidence domain** | **Study type** | **References** | **Main findings** | **Interpretation in this review** |
| --- | --- | --- | --- | --- |
| Global burden & context | Epidemiological reports | 2, 3, 5 | Rapid global rise in diabetes prevalence; strong regional and socioeconomic variation | Establishes public health relevance rather than causality |
| HFCS availability (ecological) | Ecological analyses | 4, 36 | Higher diabetes prevalence in countries with greater HFCS availability | Hypothesis-generating; confounding cannot be excluded |
| SSBs and diabetes risk | Prospective cohorts | 10, 12, 48, 54–56, 71 | Regular SSB intake linked to ~18–26% increased diabetes risk | Reflects hypercaloric intake rather than sugar chemistry alone |
| Isocaloric sugar intake | Meta-analyses | 29, 39, 53 | No consistent increase in diabetes risk under isocaloric substitution | Fructose not uniquely harmful under energy-matched conditions |
| Human hypercaloric trials | Controlled feeding | 28, 47, 49, 52 | Increased DNL, triglycerides, visceral fat, insulin resistance | Adverse effects emerge mainly with caloric excess |
| Animal models | Rodent studies | 38, 40, 44–46, 62 | Obesity, glucose intolerance, fatty liver | Mechanistic support; limited human extrapolation |
| Fructose metabolism & DNL | Mechanistic studies | 1, 21, 26, 31, 32, 41 | Fructokinase activation, phosphate depletion, enhanced DNL | Explains metabolic harm in energy-excess states |
| Endogenous fructose (polyol pathway) | Experimental animal studies | 63–65 | Polyol-derived fructose drives renal injury via fructokinase | Tissue-specific pathogenic mechanism |
| Diabetic complications | Experimental & clinical studies | 60–70 | Links to nephropathy, neuropathy, cardiomyopathy | Occurs in chronic metabolic stress contexts |
| Fruit juice vs whole fruit | Cohorts & meta-analyses | 57, 58 | Weak or no association at moderate intake | Food matrix modifies fructose effects |
| Policy & intervention | Guidelines & population studies | 6, 72, 73, 75, 81–85 | Taxation and reduced intake improve metabolic markers | Supports population-level risk reduction |
